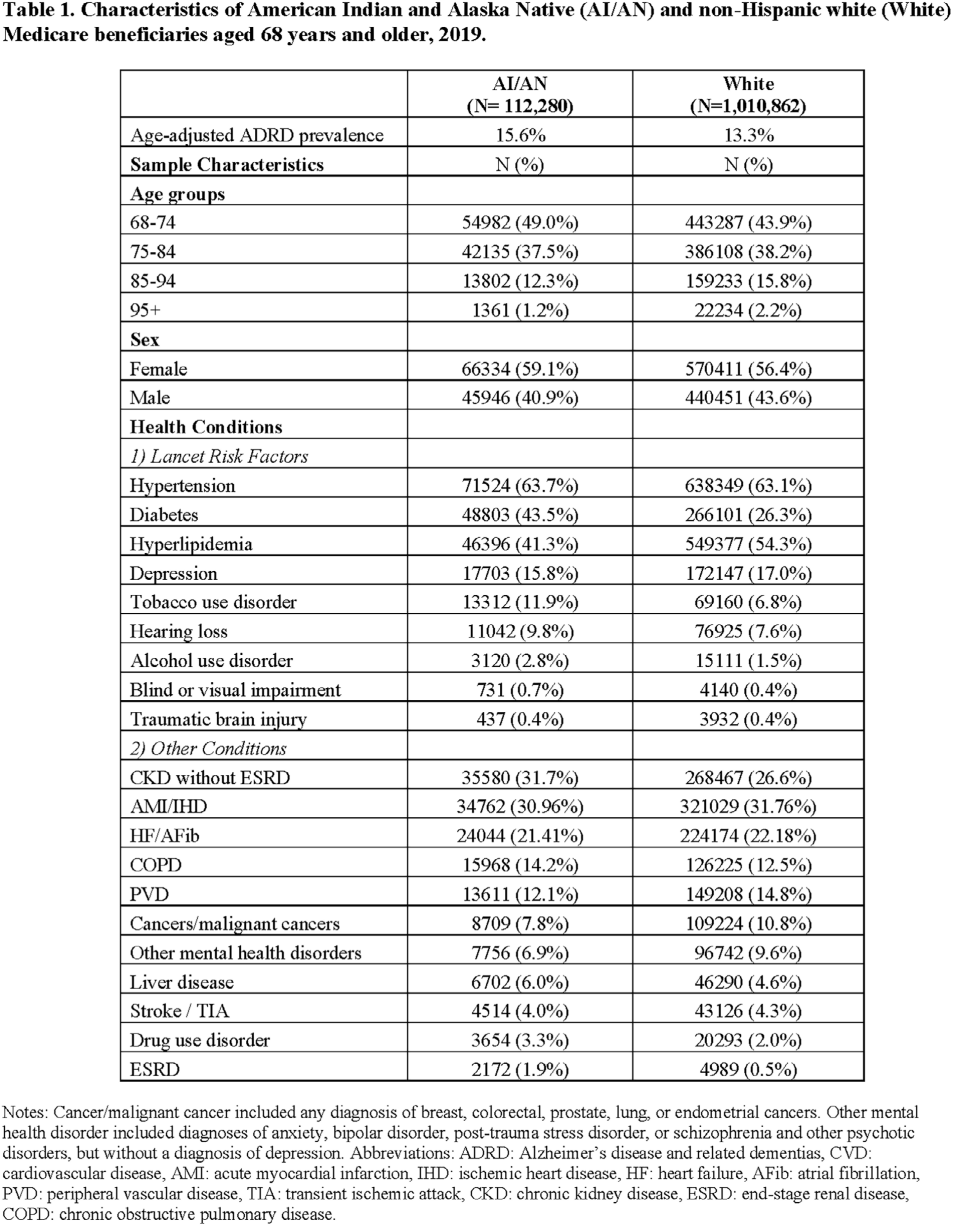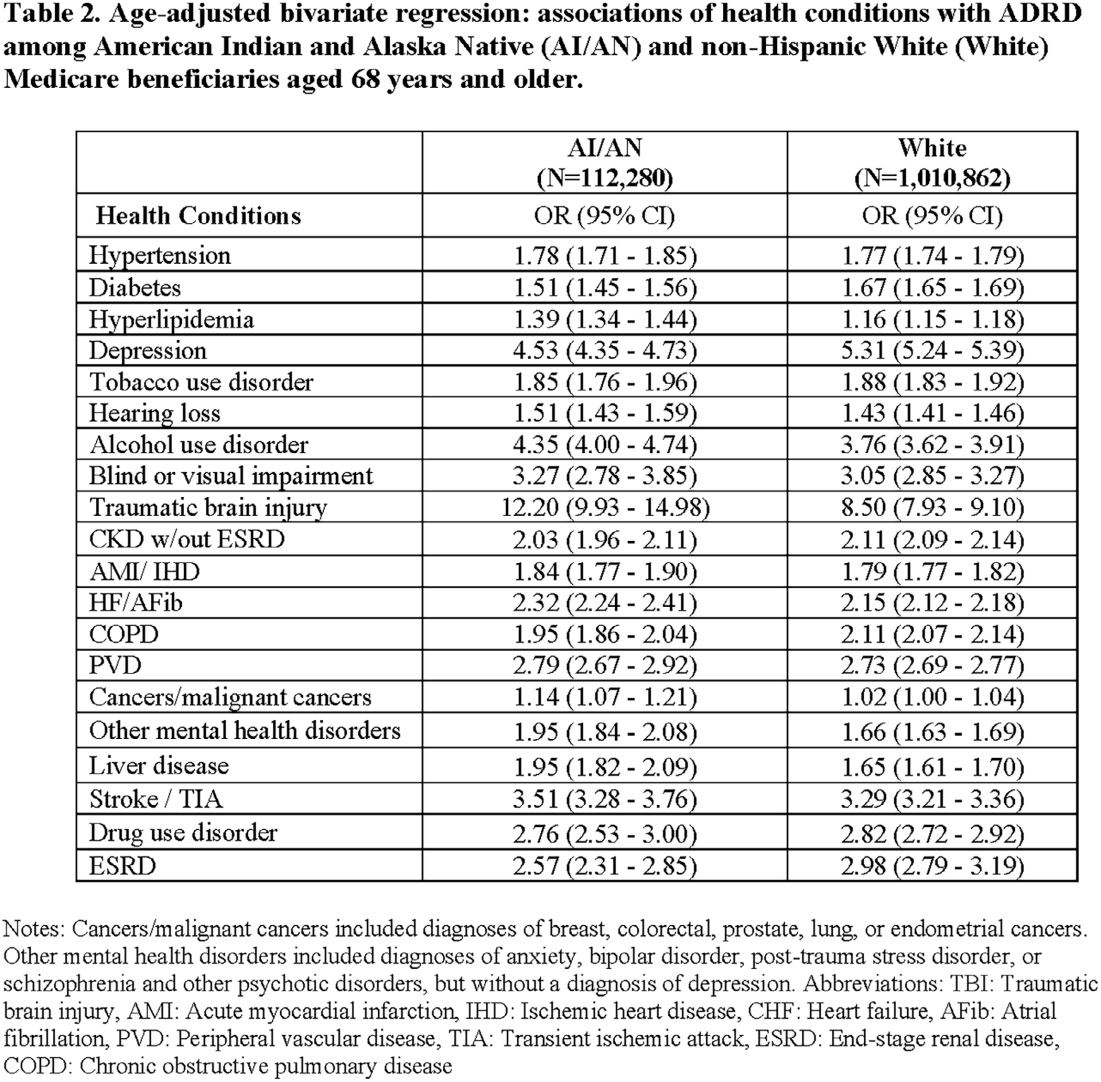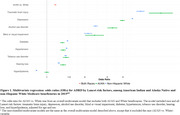# Alzheimer's Disease and Related Dementia and Related Health Conditions Among American Indian and Alaska Native Medicare Beneficiaries

**DOI:** 10.1002/alz70860_103901

**Published:** 2025-12-23

**Authors:** Manxi Yang, Ruqoyat Abdulsalam, Wenjun Fan, Spero Manson, María M. M. Corrada, Joan O'Connell, Luohua Jiang

**Affiliations:** ^1^ University of California Irvine, Irvine, CA, USA; ^2^ University of Colorado Denver, Denver, CO, USA; ^3^ University of Colorado Anschutz Medical Campus, Aurora, CO, USA; ^4^ University of California, Irvine, Irvine, CA, USA

## Abstract

**Background:**

American Indian and Alaska Native (AI/AN) peoples face a rising burden of Alzheimer's disease and related dementia (ADRD). The 2024 Lancet Commission identified modifiable risk factors for dementia for the general population; however, a comprehensive understanding of risk factors associated with ADRD among AI/AN peoples is missing. This study utilizes a national database to estimate the prevalence of health conditions associated with ADRD and examine disparities between older AI/AN and White populations.

**Method:**

We analyzed 2019 Medicare Master Beneficiary Summary File data for Medicare beneficiaries aged 68 and older, including all AI/AN beneficiaries and a 5% random sample of White beneficiaries. Two types of health conditions were examined: Lancet risk factors available in the Medicare data and other conditions that have been associated with ADRD (Table 1). Prevalence of ADRD and each condition was reported. Bivariate and multivariate logistic regressions assessed the associations between health conditions and ADRD.

**Result:**

Among 112,280 AI/AN and 1,010,862 White beneficiaries, the former had higher age‐adjusted prevalence of ADRD (15.6 % vs. 13.3%), and substantially higher prevalence of 5 out of 9 Lancet risk factors, including diabetes, alcohol use disorder (AUD), tobacco use disorder, visual impairment, and hearing loss; similar prevalence of hypertension, depression, and traumatic brain injury (TBI); but a lower prevalence of hyperlipidemia (Table 1). Bivariate analyses revealed that all conditions were associated with higher ADRD odds in both populations, with TBI, depression, and AUD showing the strongest association with ADRD (Table 2). In multivariate regressions, the associations for TBI (odds ratio (OR) = 8.00), AUD (OR=3.30), visual impairment (OR=2.38), and hearing loss (OR = 1.34) were stronger in AI/AN beneficiaries, while depression, diabetes, and hypertension had stronger associations in Whites (Figure 1).

**Conclusion:**

Older AI/AN Medicare beneficiaries compared to their White counterparts, exhibit a higher prevalence of ADRD and 5 of the 9 Lancet risk factors. Furthermore, several conditions had stronger associations with ADRD among AI/AN beneficiaries, underscoring the importance of addressing disparities in ADRD risk factors to mitigate ADRD risk among AI/AN peoples. Further research using longitudinal data with social and behavioral determinants is essential to better understand and reduce these disparities.